# Materials Contamination and Indoor Air Pollution Caused by Tar Products and Fungicidal Impregnations: Intervention Research in 2014–2019

**DOI:** 10.3390/s20154099

**Published:** 2020-07-23

**Authors:** Mateusz Kozicki, Adam Niesłochowski

**Affiliations:** Department of Thermal Physics, Acoustics and Environment, Building Research Institute, 00-611 Warsaw, Poland; a.nieslochowski@itb.pl

**Keywords:** indoor air quality, chlorophenols, chloronaphthalenes, TD-GC/MS, olfactometry, odor

## Abstract

Construction materials containing tar products are a source of indoor air pollution in buildings. This particularly concerns old buildings, in which wooden structures were impregnated with tar compositions (creosote oil and Xylamite oil containing tar products) and buildings in which bituminous seal containing hydrocarbon solvents was used. During the 1970s and 1980s, an impregnant known as Xylamite was commonly used in Polish buildings. This material still emits organic vapors into the building’s environment, significantly worsening indoor air quality (IAQ). Xylamites and other impregnating materials are a source of indoor air pollution through toxic organic compounds, such as phenol, cresols, naphthalenes, chlorophenols (CPs), and chloronaphthalenes (CNs), which emit specific odors. TD-GC/MS enables detailed identification of the reasons behind chemical indoor air pollution. The results of laboratory tests on the chemical emissions of bitumen-impregnated materials were presented in 32 case studies. In turn, the results of indoor air pollution by volatile bitumen components were presented on 11 reference rooms and 14 case studies, including residential buildings, office buildings, and others. Laboratory tests of samples of construction products confirmed the main emission sources into indoor air. The research results for the period 2014–2019 are tabulated and described in detail in this manuscript.

## 1. Introduction

Indoor air quality (IAQ) is strongly affected by volatile organic compounds (VOCs) in the air, which are emitted from building materials and furnishings. These VOCs have an adverse effect on the health and comfort of users. Research conducted by Wargocki et al. [1] shows that poor air quality has a negative effect on users who stay indoors temporarily, like office workers and students. For this reason, there is growing demand for technical devices to measure [2] and control IAQ [3]. In recent years, research activities have also been carried out in several environmental fields to investigate human odor perception and the recognition of active odor compounds [4,5,6,7,8,9]. Some studies have focused on the evaluation of the impact of odorous compounds on the quality of the indoor air environment. VOCs emitted from floor layers in residential buildings can affect indoor air quality in terms of odor annoyance, and can have other adverse effects on human health. In this context, taking into account the comfort and health of users, it is advisable that the quality of air and the internal environment is determined for the building using the research methods presented in publications [10,11].

In the second half of the 20th century, it was common in Poland to use tar-based insulation barriers and products to protect against dampness in buildings; such products included tar paper and tar adhesives. Bitumen insulation was used on the ground under the building and on the ceiling slabs of all floors, in all types of construction. For the sealing of tar paper on the joints, tar adhesives were used, first hot and then gradually replaced by cold-applied adhesives. In basements and rooms with high humidity, hydrophobic insulations were made on the basis of bitumen adhesives applied in the form of solutions in organic solvents. Bitumen binders were also used to stick wooden floors to concrete, on which tar paper was glued to level the surface.

For the protection of wood and wood-based panels against biological corrosion, products based on tar derivatives, namely creosote, and oil products known as Xylamite, were used in Poland. Xylamites were composed of a refined version of carbon oils, based on mineral oils. The main components used in Xylamites were pentachlorophenol (PCP) and other chlorophenols (CPs), chloronaphthalenes (CNs), polyphenols, a mixture of monochlorinated benzenes (MCB solvent), and residuum oils. These were usually waste materials—technical PCPs, post-distillation CNs, and regenerated CPs—used in amounts from 4% to 20% [12]. The rest of the weight percentage was fuel oil. The quantitative composition of these raw materials was varied and is not fully known.

The German equivalent of Xylamite was the wood preservative Basileum SP 70, which was used in the 1970s (1970–1981) mainly in the commercial sector for the production of chipboard and veneer in the process of mixing glue with phenol-formaldehyde resins [13]. According to the manufacturer’s instructions [14], Basileum SP70 contains 4% tributyltin oxide (TBTO) and about 80% monochloronaphthalene (CN), 15–20% dichloronaphthalene (diCN), 0.3% trichloronaphthalene (triCN), and 5% naphthalene. Other formulations were also known like Xyladecor in west Germany, Hylotox in east Germany, or Halowax series in the United States.

In the 1980s, the use of all tar-based products and processing by-products was restricted to foundations and outdoor locations. A ban on CPs, including PCP, has also been introduced in construction. Xylamite production was discontinued. Until today, some of the structures built in those years are struggling with the problem of air pollution because of the volatile components of the materials used. The most serious negative consequences were caused by the use of these products to impregnate porous fiberboards laid on the ceilings of buildings as insulation in industrial systems of the residential building industry (large panel buildings). At the time, this type of material was used in a great number of flats (their number is estimated at around 400,000). The consequences of using Xylamite in the building industry are felt to this day.

It has been found that Xylamite (mainly due to the presence of CN) has an intense, unpleasant, and musty odor that adheres to textiles, thus becoming its most characteristic feature. Users of premises where Xylamite was found complained of nose/throat irritations, burning eyes, skin irritations, and headaches. Products originating from chlorinated benzenes treated with alkali are possible sources of chlorinated dibenzo-p-dioxins (chlorinated dioxins). Technical CPs could also contain benzofurans, formed as a result of their side reactions [15]. These compounds are present in the form of numerous isomers, and are characterized by their ability to bio-accumulate in the adipose tissue of humans and animals [16,17]. The outstanding development of benzofuran derivatives for diverse diseases in a very short span of time proves its potential for medicinal chemistry research [18]. CPs, polyphenols, and CN, the main biologically active components in the discussed group of wood preservatives, are characterized by good solubility in fats and lipids, which is why they show a large affinity in the body to lipid-rich organs and nervous tissue, causing disorders of their functions and pathological changes [19,20,21,22].

Naphthalene is the most volatile member of the polycyclic aromatic hydrocarbon (PAH). It is a common air contaminant [23]. Naphthalene has received great attention since the discovery of its carcinogenicity in rats in 2000 [24]. No inference on the human carcinogenicity of naphthalene could be drawn from the International Agency for Research on Cancer (IARC) Working Group. Overall, the findings point to the fact that naphthalene might possibly be carcinogenic to humans [25]. Prolonged exposure can occur among smokers and non-smokers exposed to environmental tobacco smoke in their residences, and workers in industries where naphthalene is present at high concentrations (mothball manufacturing or creosote-impregnation facilities) [26]. In turn, CNs in indoor air have been used as a raw material for dyes and as a wood preservative with fungicidal and insecticidal properties [27]. The toxicity of CNs, like other chlorinated aromatic compounds, increases with increasing chlorination. Chlorinated naphthalenes, especially the dioxin-like congeners, have been detected in adipose tissue, liver, blood, and breast milk samples from the general population at concentration in the ng/kg lipid range [28]. Accumulation occurs in pilosebaceous acini and adipose tissue and to a lesser degree in the brain, kidneys, and other body tissues. Symptoms of chronic exposure to CNs include chloracne, cysts, headache, fatigue, vertigo, anorexia, and jaundice. This compound is a strong irritant. It may be absorbed through the skin [29]. The health effects of exposure to PCP have been summarized by the Agency for Toxic Substances and Disease Registry [30]. Long-term exposure to low levels can harm the liver, kidneys, blood, lungs, nervous system, immune system, and gastrointestinal tract. Therefore, it is essential that the concentrations of PCP in the environment are investigated.

At the European Community level, the resolution of 13 March 2019, defends clean air for everyone and highlights that people spend almost 90% of their time in indoors [31]. In many European countries, the main problem is the lack of reference standards for residential indoor air quality. In the absence of specific national references to be used for a comparison, those reported by ad hoc working groups, or in the legislation of other European countries, are currently used. Some EU Member States, such as France [32,33,34], Portugal [35], Finland [36], Austria [37], Belgium [38], Germany [39], the Netherlands [40], and Lithuania [41], have started, through a series of actions, to adopt specific guide values and reference values for pollutants, such as hydrogen cyanide, carbon monoxide, benzene, formaldehyde, trichlorethylene, tetrachloroethylene, or naphthalene. Even though the recommended guide values have no legal value, in practice, they have reached considerable importance. Settimo et al. [42] discussed and compared the different legislations present in the EU.

Legally, mandatory limits on the concentrations of substances in indoor air are still lacking in Europe. In Germany, only four indoor contaminants, such as PCBs, PCP, asbestos fibers, and tetrachloroethene, are legally regulated. The German Committee on Indoor Guide Values (AIR), formerly known as the Ad hoc Working Group (Ad hoc AG), performed health assessments of indoor air contaminants. Fromme et al. [43] includes a description of legally binding standards, indoor air guide values for substances or groups, as well as risk-related guidelines for carcinogenic substances. Polish Regulation of the Minister of Health and Social Welfare of March 12 1996 on permissible concentrations and intensities of factors harmful to health emitted by building materials, equipment and accessories in rooms intended for people [44] is in force in Poland.

This paper presents detailed studies of indoor air quality for the presence of compounds originating from wooden structures impregnated by tar compositions (creosote oil and Xylamite oil containing tar products) and bituminous seal containing hydrocarbon solvents. In order to determine the sources of VOC emissions, material samples (tar-papers, fiberboard-insulating products, wood-based construction products, and tar adhesives) were collected in the same rooms where measurements of indoor air samples were carried out, and were tested in the laboratory.

## 2. Materials and Methods

**Figure 1:**
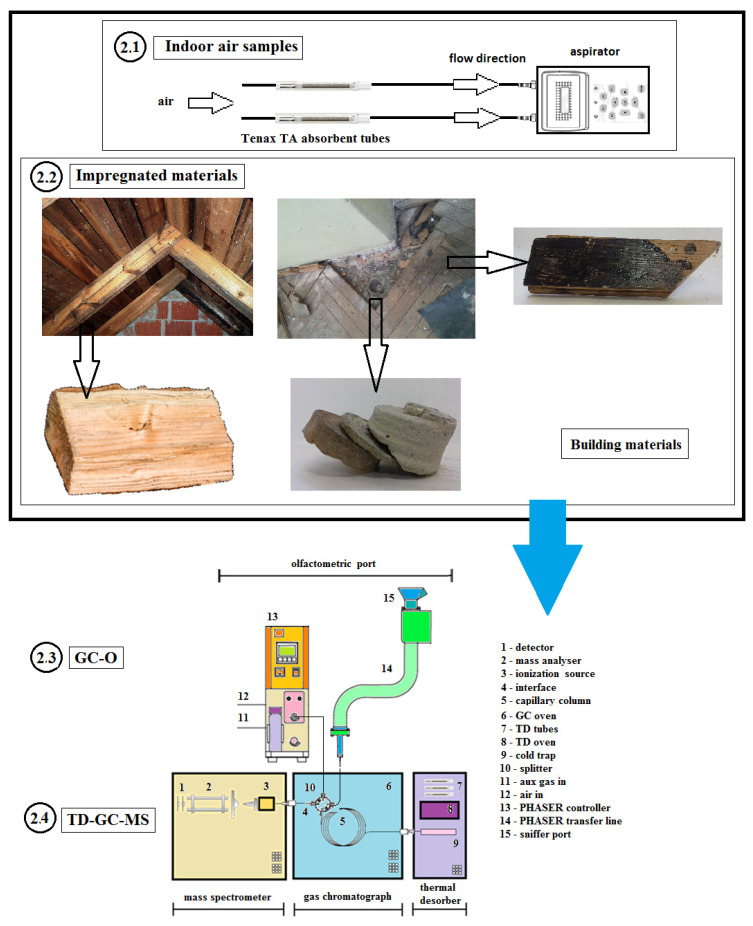
Schematic representation of the assumptions for the studies described in this publication. The individual elements of the diagram are described in detail in Section 2.1, Section 2.2, Section 2.3 and Section 2.4.

An indoor air problem was suspected in all target buildings before the research was conducted. Apartment owners, housing cooperatives, or health care units had contacted the Building Research Institute and ordered indoor air investigations concerning the suspected problems. In some cases, the party ordering the survey was able to point out a control area for the department to investigate. In other cases, the authors themselves chose the locations for sampling. Figure 1 presents a schematic representation of assumptions for the studies described in this publication. The individual elements of the diagram are described in detail in the Materials and Methods Section (Section 2.1, Section 2.2, Section 2.3 and Section 2.4).

### 2.1. Indoor Air Samples

The buildings were divided depending on their purpose and function into residential, offices, and public buildings, i.e., schools, hospitals, hotels, and museums. Between 2014 and 2019, intervention studies were carried out in 14 buildings: 4 residential, 5 offices, 3 schools, a hotel, and a museum. In total, the results were collated for 54 measuring points. The number of measuring points at each building depended on its size and the availability of places for sampling.

Indoor samples were set up in selected representative locations, approximately 1.5 m above the floor, away from windows, doors, potential emission sources, and direct sunlight. Indoor air samples were tested in accordance with ISO 16000-6: 2011 [45] and EN 16516: 2017 [46].

A dynamic method of sampling air pollutants is the Tenax TA adsorbent. The air from the building was collected onto Tenax TA tubes over a period of 1 h at a 100 mL/min air flow rate. The air flow rate was measured by means of electronic mass flow controllers, produced by Aparatura Pomiarowa Ochrony Środowiska (local manufacturer). Mass flow controllers were periodically calibrated by accredited calibration laboratories. Using the aspirators through the sorbent, 6 L of air were passed over 1 h. Flow time was measured using an electronic time meter. The chemical compounds retained on the Tenax TA sorbent were desorbed in a thermal desorber. After cryo-focusing, they were released into a carrier gas stream directly into the prepared gas chromatograph.

The measurements were conducted under naturally ventilated rooms. All doors and windows were kept closed for the 24 h preceding the measurement. Outdoor air generally contains lower concentrations of VOCs, and causes thinning of indoor air. Finally, the measurements were conducted with all doors and windows closed. The relative humidity of the measuring air ranged between 30% and 50% while the temperature range was from 18 to 24 °C.

### 2.2. Impregnated Materials Case Studies

In order to determine the sources of VOC emissions, 32 samples of insulating products (wood-based products and tar adhesive) were tested in the laboratory. These samples were cut using tools for drilling or abrasion of the surface layer. Material samples were taken in the same rooms where measurements of indoor air samples were carried out. Samples of several dozen grams were packed in clean glass jars with tight caps. Upon delivery to the laboratory, the samples underwent thermal desorption, as described in Section 2.3. The sample weight in the desorber ranged from 0.05 to 0.2 g. The desorption temperature was 80 °C and desorption time was 15 min. Helium was used as the carrier gas. The compounds were separated, identified, and quantified, as outlined in the Results and Discussion Section (Section 3).

### 2.3. GC-O Analysis

Odor analyses were carried out on a GCMS-QP2010 (Shimadzu, Tokyo, Japan) equipped with a thermal desorption TD 20 (Shimadzu, Tokyo, Japan) and coupled to the olfactory detection port (Phaser, GL Sciences, Holland). The olfactometer consisted of a glass cone purged with air previously humidified by passing through a bubbler containing water. A calculation program (Split Manager Software) calculated all flows, and split the ratio between the nose and the detector. The volatile compounds were simultaneously detected by MS and sniffing after 1:1 splitting of the eluate at the column outlet. Analyses were performed on a Restek RXI -5Sil MS capillary column (30 m × 0.25 mm ID × 0.25 µm df).

### 2.4. TD-GC-MS Analysis

The thermal desorption-gas chromatography-mass spectrometry detection technique (TD-GC-MS) was used to identify and quantify the vapors of volatile organic compounds (VOC). The vapors were identified by the electron impact (EI) mode. A Perkin Elmer Thermal Desorber TurboMatrix 350 (Liantrisant, UK) and a Perkin Elmer Clarus 500 (Shelton, USA) chromatograph were used for this purpose.

VOCs were thermally desorbed by a thermal desorber device. The tubes were heated at 300 °C and the cryo-focusing trap at −15 °C. The GC-MS detector was applied to identify VOCs. Helium was used as the carrier gas. The operating conditions of the thermodesorber were as follows: Heated valve temperature of 220 °C; transfer line temperature of 250 °C; desorption time of 15 min; desorbed helium flow of 30 mL/min; column flow of 1.0 mL/min; and injection of 100%.

The process of separation and analysis of volatile compounds was achieved using a gas chromatograph equipped with a mass spectrometer GC-MS. The following GC oven temperature program was applied: Initial temperature of 40 °C for 3 min; rate of temperature increase of 10 °C per min to 300 °C; and final oven temperature of 300 °C for 3 min. The transfer line temperature was held at 220 °C, whereas the source temperature was kept at 200 °C. Chromatographic separation was performed on a Restek RXI-5Sil MS capillary column (30 m × 0.25 mm ID × 1 μm df). The splitless injection mode was applied. The MS analysis was carried out over a scan range of 20–600 m/z with an ionization energy of 70 eV in electron ionization mode. The limit of quantification was < 1 µg/m^3^ in air.

The volatile compounds were identified by comparing the retention times of chromatographic peaks with the retention times of reference compounds, and by searching the NIST 2011 mass spectral database. All volatile compounds with mass spectra with match factors of *p* ≥ 90% were identified.

Quantitative analysis of VOCs was performed with the external standard method that uses the relationship between peak areas and the analyte concentration. The calibration curves were obtained based on six distinct known concentration levels. Standard solutions were prepared by injecting them into Tenax TA tubes placed in the inert gas flow in the GC injector. The spiked adsorbent tubes were then thermally desorbed in the same conditions as the samples.

### 2.5. Indoor Air Questionnaire

Complaints and symptoms related to the indoor environment, as experienced by the users of residential buildings, were collected from 15 flats in which indoor air and impregnated material samples were taken between 2014 and 2019 by using the indoor air questionnaire. Surveys were not carried out in the control rooms, because the users of these apartments did not complain about bad smell or health problems the source of which seemed to be their flats. Surveys were conducted only with adults. The questionnaire was developed by the authors based on interviews with residents and on various questionnaires available online [47]. The most important questions (Q1–Q8) are listed in Table 1.

Data obtained from surveys and observations made by the authors have confirmed the statements of residents. Summaries of survey data are presented in Section 3.2.

## 3. Results and Discussion

The results of research on indoor air pollutants, which have been studied extensively by the Building Research Institute for public entities and private individuals, identified the presence of over 100 substances in residential and public buildings, such as nurseries, kindergartens, and schools, across Poland. Xylenes, a mixture of isomers, 1-chloronaphthalene (1-CN), or CPs, have had the greatest impact on sanitary and health conditions over a dozen other substances, such as formaldehyde, phenol, benzene, and naphthalene.

### 3.1. Air Samples

#### 3.1.1. Rooms without Tar Impregnation Materials—Reference Rooms

Air samples were taken in 11 reference buildings: 4 residential buildings, 5 office buildings, and 2 schools. The results of the air measurements contained chemical compounds, which commonly occur in rooms used by humans. Table 2 presents the list of individual compounds and groups of compounds for rooms in which the presence of Xylamite-impregnated building materials was not found. The VOC results differ for open spaces like gymnasium and single office rooms, which may be less ventilated and more loaded with furniture.

The qualitative composition and levels of identified compounds in the indoor air were similar in all rooms tested. Volatile aldehydes (i.e., pentanal, hexanal, and higher aldehydes) and terpene hydrocarbons are compounds commonly found in wood and wood-based products. The source of aliphatic hydrocarbons and monocyclic aromatic hydrocarbons, such as toluene, can be floor products, cleaning agents, adhesives, varnish, solvents, and paints. Ethyl acetate and 1-butanol are used as solvents for paints and varnishes, and they are also a component of fragrance mixtures. Glycol ethers are used in industry as solvents for paints, varnishes (separated from varnish coatings applied on wooden surfaces), dyes, and adhesive agents, as well as components of cleaning preparations. In turn, siloxanes are emitted from wall paints, polyurethane foams, or wood care products. Simple alcohols and carboxylic acids are groups of compounds commonly found in small amounts in the air.

#### 3.1.2. Rooms with Tar-Impregnated Materials

Air samples were taken in 14 buildings: 4 residential buildings, 5 office buildings, 2 schools, a hotel, and a museum. In rooms where tar products and fungicidal impregnations were identified (Section 2.2), apart from the typical groups of chemical compounds listed in Table 2, polycyclic aromatic hydrocarbons (PAHs) and chlorinated volatile organic compounds (Cl-VOCs) were detected. Table 3 presents the results of measurements of the concentrations of bitumen and wood preservative components PAHs and Cl-VOCs in the air of buildings intended for human use.

For the clarity of data presentation, the values from individual measurements are not listed in this manuscript. Table 3 contains geometric mean (GM) values, which reflect the level of pollution diversity in different spaces with the same purpose. 

It was shown that the air in the rooms listed in Table 3 was contaminated with PAHs and vapors of Cl-VOCs. Hydrocarbons with two to four rings were identified: Naphthalene and alkyl derivatives, biphenyl, acenaphthene, dibenzofuran, fluorene, phenanthrene, anthracene, fluoranthene, and pyrene. The GM concentration of naphthalene vapors in the air was the highest in public buildings and was equal to 13 μg/m^3^ (maximum of 42 μg/m^3^), while for methylnaphthalene vapors it was 17 μg/m^3^ (maximum 34 μg/m^3^). The concentration of naphthalene vapors (GM 6 μg/m^3^, maximum of 20 μg/m^3^) and methylnaphthalenes (GM 8 μg/m^3^, maximum of 18 μg/m^3^) was slightly lower in the air of residential buildings. The lowest values were identified for office buildings: Naphthalene vapors (GM 5 μg/m^3^, maximum of 11 μg/m^3^) and methylnaphthalenes (GM 4 μg/m^3^, maximum of 18 μg/m^3^). In turn, the highest concentration of dimethylnaphthalene vapors, equal to 17 μg/m^3^ (GM 12 μg/m^3^), was recorded in old residential buildings. The main source of PAHs contamination in the air was tar paper used as insulation under the floors. Another cause of pollution was the use of Xylamite impregnation containing carbon-based oil during renovation of the buildings.

The highest concentration of acenaphthene vapors (15 μg/m^3^), dibenzofuran (16 μg/m^3^), fluorene (8 μg/m^3^), phenanthrene (20 μg/m^3^), anthracene (11 μg/m^3^), fluoranthene (3 μg/m^3^), and pyrene (1 μg/m^3^) was mainly registered in old residential buildings, and is most often associated with roof structures preserved with creosote oil.

Among the Cl-VOCs, the highest concentration of vapors in indoor air was observed for CNs, whose source is wood-based products preserved with Xylamite oil. It was observed that the higher the molecular weight of Cl-VOCs, the lower the concentration of their vapors in the air of the building. Many years of experience of both the Research Building Institute and other Polish institutions concerned with air pollution tests indicate that despite the fact that the odor is clearly felt in the premises, test results often do not show that the permitted air concentrations of the components of Xylamite, i.e., CPs, chlorobenzenes, CNs, or cresols, are exceeded. CPs as non-volatile compounds were not detected in the air in buildings in many cases; hence, we did not observe the permissible concentrations for this group of compounds being exceeded. On the other hand, chlorobenzenes, as the most volatile compounds, evaporated from the impregnated products after a year or so. The group that best reflects the described situation are CNs.

According to the Polish Regulation of the Minister of Health and Social Care of 12 March 1996 on the permitted concentrations and intensities of factors harmful to health emitted by building materials, fittings, and fixtures in premises intended for humans [44], indoor use of materials containing CPs is unacceptable. The concentration of CPs in the air of rooms intended for human stay should not exceed 15 μg/m^3^ in category A premises and 20 μg/m^3^ in category B premises, whereas the concentration of CNs should not exceed 15 and 30 μg/m^3^, respectively. Category A premises are residential premises, premises for permanent residence of the ill in health care institutions and for permanent residence of children and youth in educational buildings, as well as premises intended for storing food products. In turn, category B premises are all other premises, such as premises intended for people in public buildings, other than those included in A category premises, and auxiliary premises. The permissible concentration of CNs according to the Polish Regulation of the Minister of Health and Social Care [44] was exceeded in 6 out of 14 cases. In turn, the permissible concentration of CPs was not exceeded in any of the cases examined. It is assumed that even if the concentration of CPs and CNs in the air do not exceed the permissible level, the intense characteristic odor of Xylamite is reason enough for carrying out the renovation combined with the drying of impregnated materials.

In some cases, the concentration of the examined compounds was very high and exceeded the permitted norms by many times; in others, it was low, even on the limit of detection. However, in all of the tested flats, there was the odor characteristic of preparations from the Xylamite group, and only its intensity differed (Section 3.4).

**Figure 2:**
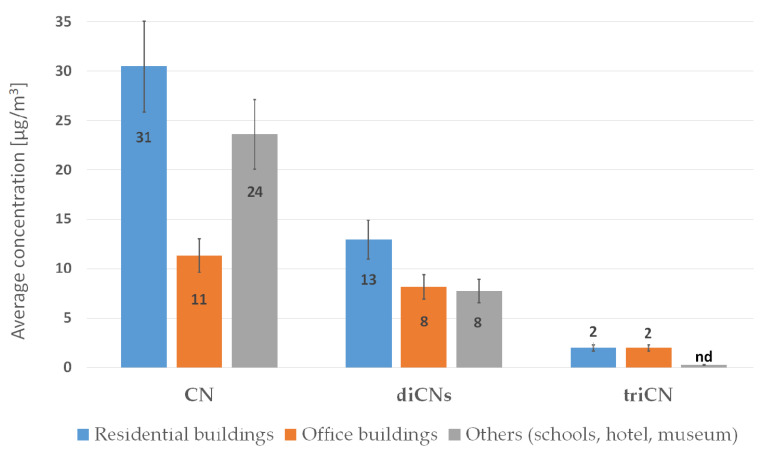
GM values for Cl-VOC concentrations in the indoor air of buildings intended for human habitation, for which users had complained about poor air quality.

The range of Cl-VOC concentrations emitted from construction products containing bitumen and Xylamite is shown in Figure 2.

The presence of naphthalene and CN compounds in measurements of air quality indicates contamination with tar products and impregnants based on tar products, which was confirmed by the results of material tests measured in these rooms (Section 3.3). Naphthalene was identified in the air of residential buildings at all investigated points (12/12), in office buildings at 17 out of 24 points, and in public buildings (schools, hotel, museum) at 16 out of 18 points. In turn, CNs were identified in residential buildings at 10 out of 12 points, while in office and public buildings at all investigated points.

An increase in the number of methyl and chlorine derivatives of naphthalene causes the amount of vapors of these compounds in indoor air to decrease. This is related to the different contents of these derivatives in impregnation materials and also to their different volatilities. For example, the boiling point for 1-CN is 259 °C; for 1,4-diCN, it is 288 °C; and for 1,3,7-triCN, it is 309 °C.

### 3.2. Health Symptoms

The investigated apartments were not chosen at random; an indoor air problem was suspected in each one prior to the survey. On every occasion, the reason behind the intervention research undertaken was users’ complaints about difficult hygienic conditions and, particularly, chemical smells. The assessments were further prompted by general interventions, renovations, upgrades, and greater public awareness. It is noteworthy that the chemical odors continued to be perceptible, despite the fact that over 40 years had passed since the original impregnation.

In the questionnaire (Section 2.5), users were asked to recall environmental problems and associated symptoms that had persisted for years that became permanent. The authors collected this data focusing on recurring symptoms attributed to the home environment.

A list of health symptoms described by users of 15 residential buildings is summarized in Table 4. Over half of the survey participants complained about (Q1) mucous membrane eye irritation (12/15), cough (13/15), headache and malaise (13/15), and fatigue (12/15). Survey participants also reported conditions, such as nasal symptoms (5/15), wheezing (6/15), asthma (4/15), and allergic skin reactions (4/15). Those who declared the latter two also highlighted that they had been formally diagnosed by specialists. Additionally, users also complained about nausea, irritation of the respiratory tract, and eye, nose, and throat discomfort. None reported loss of coordination and dizziness. It is important to underline that each of the respondents indicated that their flatmates complained about similar problems (Q5).

Based on the results obtained from the questionnaires, it can be concluded that the health problems of residents had either been going on for a long time (several dozen years) or since moving into flats where there was a problem with Xylamite (Q3). Residents unanimously declared that health symptoms occurred regardless of the time of day/year (Q2). Some respondents (4/15) had noticed a correlation between the increase in temperature and humidity and the exacerbation of symptoms, also during wintertime (Q4). More symptoms appeared when the room temperature was higher. This could point to a temperature dependence of the VOC emissions from the tar-based building materials. The percentage of smokers among the residents in the survey was low (2/15). None smoked inside the apartment (Q7). Further, mold was absent from all examined flats (Q8). The apartments were ventilated every day by all respondents (Q6). The questionnaires were addressed to adults only. The complaints concerning environmental factors did not differ among age and gender groups.

The above evidence suggests that tar products and fungicidal impregnations used in the 1970s had caused an indoor air problem. The complaints and symptoms reported by the tested group, however, do not provide a reliable overall picture, as the number of individuals voicing their opinion is not large enough. Nonetheless, taking into account the numerous interviews with residents (sometimes confirmed by specialists) and the results of surveys, it is suggested that the situation is worrying. For decades, the Building Research Institute has been receiving hundreds of complaints from the users of their premises, who said that specific chemical odors were present in the flats, which caused a series of symptoms of ill-being.

The chemical pollutants and indoor air quality data results gathered by the Building Research Institute over the years 1986–1997 were used to assess health risks in the context of the physiological development of children. Czernych et al. [48] conducted a toxicity assessment for indoor VOCs, based on the available scientific data, to gauge the adverse effects on exposed children and adolescents. The diverse sources of VOC emissions, our limited knowledge on the additive effects of various compounds, and the effects of prolonged exposure at relatively low concentrations hinder our understanding of the health effects of poor air quality [20].

### 3.3. Samples of Construction Products

This section presents the results of laboratory tests for the emission of VOCs taken from samples of construction products in buildings, where users had complained about poor hygienic conditions (chemical smells, overall poor health). In total, over the years 2014–2019, samples for this study were collected from 32 different premises intended for human habitation.

**Figure 3:**
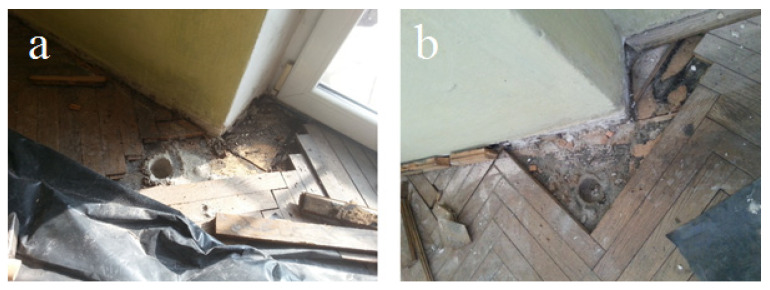
Floor material sampling points in two different (**a**) and (**b**) residential buildings (photos taken by the authors).

**Figure 4:**
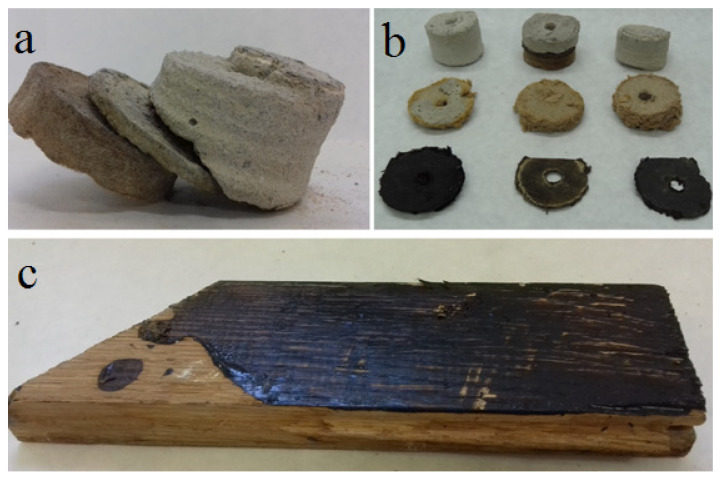
Samples of materials from floor layers in residential buildings: (**a**) concrete floor slab + tar adhesive + soft insulating fiberboard from a single and (**b**) different sampling points; (**c**) wooden construction covered by tar adhesive (photos taken by the authors).

Samples of the following building products were tested in the laboratory: soft insulation fiberboard, chemically preserved wooden construction elements, tar paper, and tar adhesives (Figure 3 and Figure 4). The size of the collected samples was equal to several dozen grams. The test was performed using a thermal desorber at a temperature of 80 °C, as described in the Materials and Methods Section (Section 2). The temperature was set at such a level that, on the one hand, the test could allow the desorption of the greatest amount of the compounds from the samples tested and, on the other hand, that it would not cause the product to be destroyed. It was desirable that the result would in the best way indicate the content of VOCs in the products. The main assumption was to check whether the tested products consisted of chemicals detected in the air. These tests allowed the desorption of the greatest amount of compounds from the samples tested and a comparison of them with compounds adsorbed on a Tenax TA at room temperature. The Supplementary Material presents the results of VOC emission measurements from samples of construction products taken from different buildings. The results of the measurements were presented in units of mg/kg of the sample mass (or different μg/g sample weight).

The source of emission of PAHs was bituminous materials containing tar insulation paper, tar adhesives, and wood preservatives—creosote and products under the trade name Xylamite. The source of CN and CP emissions was also Xylamite. If the insulation paper or tar adhesive emitted Cl-VOCs, this was the result of the incorporation of Xylamite, a porous fiberboard or wood close to the material, as a preservative.

The following paragraph describes in detail 4 specific cases out of 21 collected in Table 4. Xylamite-impregnated porous fiberboard applied under floors in one of the public building emitted methylnaphthalenes up to 244 mg/kg, dimethylnaphthalenes up to 732 mg/kg, acenaphthene up to 484 mg/kg, and dibenzofuran up to 263 mg/kg. Wooden roof elements conserved with creosote emitted methylnaphthalenes in the amount up to 244 mg/kg, dimethylnaphthalenes to 377 mg/kg, acenaphthene to 270 mg/kg, fluorene to 240 mg/kg, phenanthrene to 619 mg/kg, anthracene to 541 mg/kg, phenol up to 31 mg/kg, xylenols up to 52 mg/kg, benzothiophene up to 13 mg/kg, and quinoline up to 67 mg/kg. Wood samples from the roof structure in another residential building emitted methylnaphthalenes in an amount up to 258 mg/kg, dimethylnaphthalene up to 132 mg/kg, acenaphthene up to 100 mg/kg, cresols up to 92 mg/kg, xylenols up to 146 mg/kg, benzothiophene up to 3 mg/kg, and 2-methylobiphenyl up to 26 mg/kg. In turn, tar paper used as underfloor insulation emitted naphthalene in an amount up to 25 mg/kg, methylnaphthalenes up to 65 mg/kg, dimethylnaphthalenes up to 84 mg/kg, acenaphthene up to 37 mg/kg, fluorene up to 16 mg/kg, phenanthrene up to 8 mg/kg, dibenzofuran up to 24 mg/kg, cresols up to 41 mg/kg, and xylenols up to 72 mg/kg.

The content of CNs and CPs in construction products is the result of preserving wood and porous fiberboards with Xylamite oil. The highest emission level of CN equal to 696 mg/kg was observed for tar paper. Insulation fiberboard embedded in contact with the tar paper emitted CNs up to 176 mg/kg. The highest emissions of diCNs (up to 34 mg/kg) and triCNs (up to 65 mg/kg) were observed in the samples of wood boards embedded as a ceiling in an old residential building. This wood also emitted 2,4-diCP in an amount up to 32 mg/kg.

**Figure 5:**
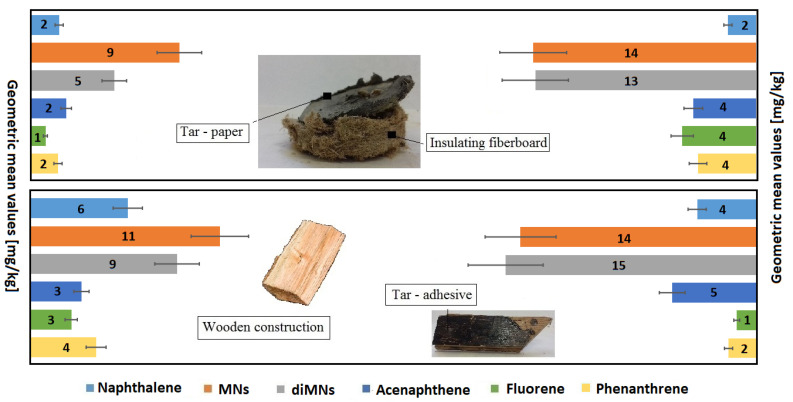
Emission of PAHs from samples of construction products taken from buildings intended for human habitation, where complaints about unacceptable hygienic conditions were raised.

**Figure 6:**
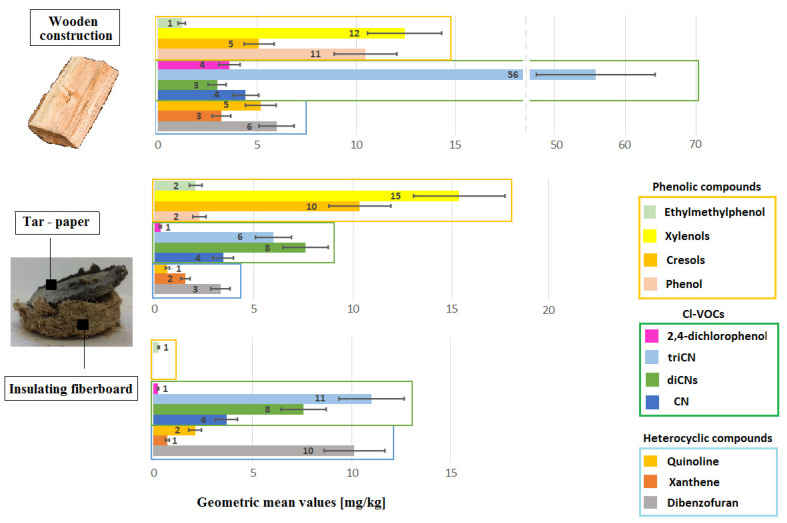
Emission of heterocyclic aromatic hydrocarbons, organochlorine derivatives, and phenolic compounds from samples of construction products taken from buildings intended for human habitation, where complaints about unacceptable hygienic conditions were raised.

Figure 5 and Figure 6 graphically show the emission levels of VOCs from construction products taken from the buildings in question (Section 2.2 and Supplementary Material).

The results for tar adhesives are not shown in Figure 6, because this group only emits PAH compounds, which originate from poorly cleaned asphalt. Tar adhesives do not emit heterocyclic, Cl-VOCs, and phenolic compounds. CN and cresols, which were identified in small quantities, are secondary contaminants originating in neighboring floor construction materials containing Xylamite.

Increasing the temperature increases the molecules’ speed. Higher temperatures usually result in higher diffusion and thus emissions [49,50]. The chemical emission for some Cl-VOCs was measured at a series of temperatures spanning from 80 to 130 °C, so at temperatures well in excess of room temperature. This test allowed the desorption of the greatest amount of compounds from the samples tested and a comparison of them with compounds adsorbed on a Tenax TA at room temperature. Table 5 presents the results of the emission test from wood samples impregnated with Xylamite. Wood samples were taken from a wooden recreational house built in the 1970s, which was one of the 32 premises collected in the Supplementary Material. The indoor air inside the house was contaminated with 1-CN and diCNs, which was confirmed by the measurements (Section 3.1.2). CPs as non-volatile compounds were not detected at room temperature in the air in buildings in many cases. In turn, chlorobenzenes, as the most volatile compounds, evaporated from the impregnated products many years ago. This is another argument that the group that best reflects the described situation are CNs.

The emissions of all detected compounds increased with elevating temperature. The amount and composition of Cl-VOCs were markedly influenced by changes in temperature. In wood samples of temperatures of 80 °C, only small amounts of 1-CN and diCN were emitted. This information is sufficient to check whether the tested products consisted of chemicals detected in the air. Larger substituent number Cl-VOCs were above detection at 100 °C. After increasing the desorption temperature, pairs of triCP, tetraCP, and PCP gradually began to be released from the wood. The most suitable technique for determining PCP at this level is gas chromatography with electron capture detection (GC-ECD) [51].

### 3.4. Identification of Odors from Construction Products by GC/MS-O

Xylamite is characterized by a very strong and specific chemical odor. This odor was described by the producer as ‘non-lasting’, which among other reasons encouraged its use in the residential building industry. Today, 40 years since the last application, we can say that the smell is practically everlasting. The characteristic odor of these preservatives is clearly felt in the premises and is a great nuisance for dwellers to this day. It seems that the emission rates decrease slightly over time and there is significant surface uptake of the compounds.

**Figure 7:**
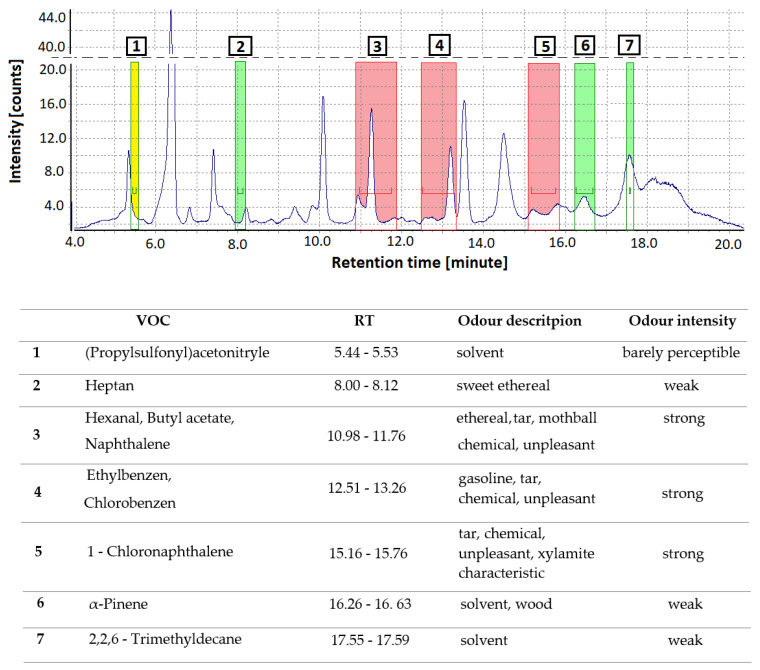
GC-MS chromatogram with olfactory detection port signals, categorizing the intensity of odors as “barely perceptible” (yellow on the spectrum), “weak” (green), and “strong” (red). The table reports the identified odor signals according to the peak number.

GC/MS-O was used to identify seven odor-active compounds in the Xylamite-impregnated wooden floor samples (Figure 7). The GC-MS/O methodology allows the establishment of a correlation between the chemical nature and concentration of specific odor compounds and the human perception of smell. Each of the perceived compounds was identified based on the retention time, reference compounds, and by searching the NIST 2011 mass spectral database. Odorants were described by four panelists, in their own words, according to their previous experience. No anosmia was reported. The experts also evaluated the odor intensity scale, with naphthalene, chlorobenzene, and 1-CN being the odorants perceived to have the strongest intensity. Naphthalene had an ethereal, tar, mothball, and strong chemical smell; chlorobenzene had a gasoline and tar smell; whereas 1-CN had a strong, unpleasant, tar, chemical, and Xylamite-characteristic odor. Heptane (sweet ethereal), α-pinene (solvent, wood), and 2,2,6-trimethyldecane (solvent) were odor-active compounds with a weak intensity.

## 4. Conclusions

The introduction of Xylamite in the building industry had serious economic consequences tied in with the costs associated to renovations lasting to this day and financed initially by the state budget and the owners of the buildings. The health effects for dwellers of such buildings in which fiberboards impregnated with Xylamite were used seem to be much more serious. For tens of years, some residents have been in contact with the toxic volatile components of Xylamite. Despite the passing of almost 30 years since the ban on using Xylamite in the residential building industry in Poland, there continues to be an unknown number of buildings containing materials impregnated with Xylamite.

The air pollution tests were examined in places where impregnated materials were found. It should be pointed out that the presence of harmful compounds was always detected in the air. This shows that the users of these flats had for many years been in contact with polluted air. Symptoms associated with indoor air problems were common in all the surveyed flats, which was confirmed by the results of the Indoor Air Questionnaire. Air pollution tests carried out in old buildings constructed or renovated in the second half of the 20th century have shown that they may be contaminated with chemical compounds, whose sources are tar-based products and impregnations used for wood and wood-based structures with chloroorganic compounds. The main constituents of the contaminants were identified as PAHs, CNs, and CPs. Laboratory tests of samples of construction products confirmed the main emission sources and determined to what extent products can affect air pollution.

In laboratory conditions, PCP was emitted from impregnated wood only at temperatures above 120 °C. This justifies the earlier observation that the air inside the buildings in question is not contaminated by PCP vapours or their concentrations are below the method’s detection level at 120 °C. The permissible concentration of CNs according to the Polish Regulation of the Minister of Health and Social Care was exceeded in 6 out of 14 cases. CPs, as non-volatile compounds, were not detected in the air in buildings in many cases; hence, we did not observe the permissible concentrations for this group of compounds being exceeded. In turn, chlorobenzenes, as the most volatile compounds, evaporated from the impregnated products many years ago. Today, it is generally accepted that the detection of chlorinated aromatic compounds in building material, air, or dust automatically triggers remedial measures. Thus, it is advisable to remove the floor layers, which contain reported compounds.

The GC/MS-O methodology can be a useful tool for recognizing odor-active compounds, which are characteristic of Xylamite products, and to relate qualitative and quantitative information that conforms to human perception. Naphthalene, chlorobenzene, and 1-CN proved to be responsible for the overall odor annoyance perceived as coming from tar products and fungicidal impregnations.

The example of Xylamite shows how important it is to evaluate the properties of building materials with respect to their chemical composition. Products based on chemical raw materials should undergo evaluations based on emission tests or examinations for harmful substance contents.

## Figures and Tables

**Table 1:** Questions presented to people permanently residing in apartments, where there was a problem with Xylamite.

Questions
What kinds of symptoms or discomforts are you experiencing that you feel are related to the home environment? *Please check each symptom or adverse health effect you experience more than three times per week over the span of a year.* Options to choose from: mucous membrane eye irritation, cough, headache and malaise, fatigue, asthma, nasal symptoms, wheezing, loss of coordination, dizziness, allergic skin reaction. Have these symptoms been diagnosed by a general practitioner or specialist?
2. When do the health problems occur? Options to choose from: morning, afternoon, all day, no noticeable trend Do the problems occur more frequently during specific seasons of the year?
3. When have these complaints started?
4. Have you noticed any other events (such as weather events, temperature or humidity changes) that tend to occur at about the same time as your symptoms?
5. Do any of your flatmates complain about similar problems?
6. Do you often open windows for ventilation?
7. Do you smoke? Do you smoke inside the property?
8. Are there any areas inside the house where mould appears?

**Table 2:** Concentration of vapors of VOCs in the indoor air of buildings. The results were collated for 56 measuring points in 11 reference buildings, which were divided according to their purpose and function.

Identified Chemical Compounds [CAS Number]	Concentration of Vapors in Indoor Air [μg/m^3^]								
	Residential Buildings (4 Cases, 16 Points)			Office Buildings (5 Cases, 28 Points)			Public Buildings (Schools) (2 Cases, 12 Points)		
	**N**	**max.**	**GM**	**N**	**max.**	**GM**	**N**	**max**	**GM**
1-Butanol [71-36-3]	16	42 ± 6	13 ± 2	28	53 ± 8	4 ± 1	10	5 ± 1	3 ± 1
Toluene [108-88-3]	16	52 ± 8	20 ± 3	28	21 ± 3	5 ± 1	12	14 ± 2	6 ± 1
Xylene, mixed isomers [1330-20-7]	10	27 ± 4	16 ± 2	28	34 ± 5	6 ± 1	12	18 ± 3	5 ± 1
Ethyl Acetate [141-78-6]	16	43 ± 6	17 ± 3	18	12 ± 2	3 ± 1	6	4 ± 1	2 ± 1
Aliphatic Hydrocarbons C6 - C16 [v]	16	159 ± 24	65 ± 10	28	70 ± 11	26 ± 4	12	24 ± 4	12 ± 2
Alcohols C3 - C10 [v]	16	33 ± 5	22 ± 3	22	33 ± 5	9 ± 1	12	66 ± 10	62 ± 9
Siloxanes and Cyclosiloxanes [v]	12	78 ± 12	40 ± 6	24	79 ± 12	22 ± 3	12	59 ± 9	30 ± 5
Carboxylic Acids C2 - C12 [v]	14	15 ± 2	10 ± 2	16	11 ± 2	8 ± 1	12	6 ± 1	4 ± 1
Aldehydes C5 - C10 [v]	16	167 ± 25	56 ± 8	28	68 ± 10	30 ± 5	12	47 ± 7	19 ± 3
Terpene Hydrocarbons C6 - C16 [v]	12	53 ± 8	18 ± 3	10	23 ± 3	9 ± 1	10	7 ± 1	4 ± 1
Glycols and Glycols Alkyl Ethers [v]	12	84 ± 13	17 ± 3	18	11 ± 2	7 ± 1	12	46 ± 7	27 ± 4

The expanded uncertainty representing the 95% confidence interval is 15%. The expanded uncertainty was calculated using a factor of k = 2. The uncertainty of the results was estimated on the basis of available data, including data on the accuracy of the measurement system used and data on repeatability obtained experimentally. N: number of measurements in which a given compound was identified. max.: the highest identified value. GM: geometric mean value for all measurements. [v]: number of compounds identified is different for each measuring points; values are the sums of all the compounds belonging to the group.

**Table 3:** Concentration of vapors of PAHs and Cl-VOCs in the indoor air of buildings where tar products and Xylamites were identified. The results were collated for 54 measuring points in 14 buildings, which were divided according to their purpose and function.

Identified Chemical Compounds [CAS Number]	Concentration of Vapors in Indoor air [μg/m^3^]								
	Residential Buildings (4 Cases, 12 Points)			Office Buildings (5 Cases, 24 Points)			Public Buildings (Schools, Hotel, Museum) (5 Cases, 18 Points)		
**PAHs**									
	**N**	**max.**	**GM**	**N**	**max.**	**GM**	**N**	**max.**	**GM**
Naphthalene [91-20-3]	12	20 ± 3	6 ± 1	17	11 ± 2	5 ± 1	16	42 ± 6	13 ± 2
Methylnaphthalenes [91-57-6, 90-12-0]	9	18 ± 3	8 ± 1	17	18 ± 3	4 ± 1	8	34 ± 5	17 ± 3
Dimethylnaphthalenes [575-43-9, 582-16-1, 571-61-9, 569-41-5]	6	17 ± 3	12 ± 2	7	12 ± 2	3 ± 1	7	8 ± 1	5 ± 1
Biphenyl [92-52-4]	4	4 ± 1	4 ± 1	4	4 ± 1	3 ± 1	3	3 ± 1	2 ± 1
Acenaphthene [83-32-9]	8	15 ± 2	7 ± 1	5	1 ± 1	1 ± 1	5	3 ± 1	2 ± 1
Dibenzofuran [132-64-9]	8	16 ± 2	7 ± 1	7	4 ± 1	2 ± 1	3	4 ± 1	2 ± 1
Fluorene [86-73-7]	6	8 ± 1	3 ± 1	nd			nd		
Phenanthrene [85-01-8]	8	20 ± 3	3 ± 1	1	1 ± 1	1 ± 1	nd		
Anthracene [120-12-7]	4	11 ± 2	4 ± 1	nd			nd		
Fluoranthene [206-44-0]	4	3 ± 1	2 ± 1	nd			nd		
Pyrene [129-00-0]	1	4 ± 1	1 ± 1	nd			nd		
**Cl-VOCs**									
Chloronaphthalene [90-13-1]	10	121 ± 18	31 ± 5	24	40 ± 6	11 ± 2	18	215 ± 32	24 ± 4
Dichloronaphthalenes [2050-75-1, 2198-77-8]	8	56 ± 8	13 ± 2	13	20 ± 3	8 ± 1	15	109 ± 16	8 ± 1
Trichloronaphthalene [55720-37-1]	2	2 ± 1	2 ± 1	9	5 ± 1	2 ± 1	nd		
Dichlorophenol [120-83-2]	2	2 ± 1	2 ± 1	nd			nd		

The expanded uncertainty representing the 95% confidence interval is 15%. The expanded uncertainty was calculated using a factor of k = 2. The uncertainty of the results was estimated on the basis of the available data, including data on the accuracy of the measurement system used and data on repeatability obtained experimentally. N: number of measurements in which a given compound was identified. max.: the highest identified value. GM: geometric mean value for all measurements. nd: not detected.

**Table 4:** List of health symptoms described by users of 15 residential buildings in which Xylamite was identified.

Health Symptoms	No Syndrome	Sporadic (below 7 Cases)	Frequent (above 7 Cases)
mucous membrane eye irritation			x
cough			x
headache and malaise			x
fatigue			x
wheezing		x	
asthma		x	
nasal symptoms		x	
allergic skin reaction		x	
loss of coordination, dizziness	x		

**Table 5:** Emission of VOCs from wood samples impregnated with Xylamite, taken from a recreational house.

Desorption Temperature [°C]	Cl-VOCs [μg/g]				
	1-CN	diCN	triCP	tetraCP	PCP
80	0.21 ± 0.03	0.13 ± 0.02	–	–	–
90	0.24 ± 0.04	0.12 ± 0.02	–	–	–
100	0.19 ± 0.03	0.17 ± 0.03	0.05 ± 0.01	–	–
110	1.26 ± 0.19	0.88 ± 0.13	0.03 ± 0.01	0.77 ± 0.12	–
120	4.23 ± 0.63	3.70 ± 0.11	2.05 ± 0.31	3.48 ± 0.52	2.00 ± 0.30
130	3.80 ± 0.11	3.72 ± 0.11	2.72 ± 0.41	5.53 ± 0.83	3.44 ± 0.52

The expanded uncertainty representing the 95% confidence interval is 15%. The expanded uncertainty was calculated using a factor of k = 2. The uncertainty of the results was estimated on the basis of available data, including data on the accuracy of the measurement system used and data on repeatability obtained experimentally.

## Supplementary Materials

The following are available online at https://www.mdpi.com/1424-8220/20/15/4099/s1, Table S1: Table VOC emission from samples of construction products taken from different buildings. In total, 73 samples were taken from 32 premises.
