# Supplementary material for: Materials Contamination and Indoor Air Pollution Caused by Tar Products and Fungicidal Impregnations: Intervention Research in 2014–2019
Source: Sensors (Basel). 2020 Jul 23;20(15):4099. doi: 10.3390/s20154099 (PMC7435637; doi:10.3390/s20154099)
Supplement: Supplementary file 1 [file sensors-20-04099-s001.pdf]

sensors

MDPI

Supplementary Material

Materials Contamination and Indoor Air Pollution Caused by Tar Products and Fungicidal Impregnations: Intervention Research in 2014–2019

Mateusz Kozicki * and Adam Niesłochowski

Department of Thermal Physics, Acoustics and Environment, Building Research Institute, 00-611 Warsaw, Poland; a.nieslochowski@itb.pl

* Correspondence: m.kozicki@itb.pl; Tel.: +48-22-57-96-187

Received: 17 June 2020; Accepted: 20 July 2020; Published: date

Table S1. Table VOC emission from samples of construction products taken from different buildings. In total, 73 samples were taken from 32 premises.

Identified Chemical Compound [CAS Number]	The Mass of the Separated Chemical Compound Calculated in Milligrams Per Kilogram of Sample Mass [mg/kg]											
	Tar Paper (11 Cases; 25 Samples)			Insulating Fiberboard (10 Cases; 16 Samples)			Wooden Construction (7 Cases; 26 Samples)			Tar Adhesive (4 Cases; 6 Samples)		
	PAHs											
	N	max.	GM	N	max.	GM	N	max.	GM	N	max.	GM
Naphthalene [91-20-3]	19	25 ± 4	2 ± 1	13	31 ± 5	2 ± 1	25	206 ± 31	6 ± 1	6	38 ± 6	4 ± 1
Methylnaphthalenes [91-57-6, 90-12-0]	17	65 ± 10	9 ± 1	11	244 ± 37	14 ± 2	21	258 ± 39	11 ± 2	4	21 ± 3	14 ± 2
Dimethylnaphthalenes [575-43-9, 582-16-1, 571-61-9, 569-41-5]	13	84 ± 13	5 ± 1	8	732 ± 110	13 ± 2	21	377 ± 57	9 ± 1	3	21 ± 3	15 ± 2
Trimethylnaphthalenes [829-26-5, 2131-41-1]	7	25 ± 4	1 ± 1	2	3 ± 1	2 ± 1	13	95 ± 14	5 ± 1		nd	
Biphenyl [92-52-4]	10	16 ± 2	2 ± 1	6	4 ± 1	1 ± 1	21	87 ± 13	3 ± 1	3	6 ± 1	5 ± 1
2-Methylobiphenyl [643-58-3]	2	24 ± 4	4 ± 1	2	76 ± 11	47 ± 11	8	56 ± 8	2 ± 1		nd	
Acenaphthylene [208-96-8]	1	3 ± 1	3 ± 1	2	190 ± 28	126 ± 19	1	21 ± 3	21 ± 3		nd	
Acenaphthene [83-32-9]	13	37 ± 6	2 ± 1	10	484 ± 73	4 ± 1	22	270 ± 40	3 ± 1	3	6 ± 1	5 ± 1
Fluorene [86-73-7]	11	16 ± 2	1 ± 1	5	170 ± 26	5 ± 1	20	240 ± 36		4	4 ± 1	1 ± 1
2-Hydroxyfluorene [2443-58-5]		nd			nd		14	104 ± 16	15 ± 2		nd	
9H-Fluoren-9-on [486-25-9]	1	3 ± 1	3 ± 1	2	39 ± 6	28 ± 4	1	1 ± 1	1 ± 1		nd	
Phenanthrene [85-01-8]	15	8 ± 1	2 ± 1	12	81 ± 12	4 ± 1	22	619 ± 93	4 ± 1	4	5 ± 1	2 ± 1
Anthracene [120-12-7]	6	4 ± 1	1 ± 1	2	8 ± 1	7 ± 1	16	541 ± 81	22 ± 3	3	2 ± 1	1 ± 1
Methylanthracenes [610-48-0, 613-12-7]		nd			nd		2	7 ± 1	7 ± 1		nd	
Fluoranthene [206-44-0]		nd			nd		15	57 ± 9	6 ± 1		nd	
Pyrene [129-00-0]	3	1 ± 1	1 ± 1	2	1 ± 1	1 ± 1	15	35 ± 5	3 ± 1		nd	
	Heterocyclic compounds											

Dibenzofuran [132-64-9]	13	23 ± 3	3 ± 1	7	263 ± 39	10 ± 2	19	258 ± 39	6 ± 1		nd	
9H-Xanthene [92-83-1]	6	6 ± 1	2 ± 1	2	1 ± 1	1 ± 1	16	98 ± 15	3 ± 1		nd	
Benzothiophene [270-82-6]	2	1 ± 1	1 ± 1		nd		16	13 ± 2	2 ± 1		nd	
2-Methylpyridine [109-06-8]		nd		2	3 ± 1	3 ± 1		nd			nd	
Quinoline [91-22-5]	3	1 ± 1	1 ± 1	5	22 ± 3	2 ± 1	11	67 ± 10	5 ± 1		nd	
**Cl-VOCs**												
Chloronaphthalene [90-13-1]	10	696 ± 104	4 ± 1	16	176 ± 26	4 ± 1	12	32 ± 5	4 ± 1	1	2 ± 1	2 ± 1
Dichloronaphthalenes [2050-75-1, 2198-77-8]	3	12 ± 2	8 ± 1	6	24 ± 4	8 ± 1	12	34 ± 5	3 ± 1		nd	
Trichloronaphthalene [55720-37-1]	3	8 ± 1	6 ± 1	3	15 ± 2	11 ± 2	2	65 ± 10	56 ± 8		nd	
2,4-Dichlorophenol [120-83-2]	1	1 ± 1	1 ± 1	1	1 ± 1	1 ± 1	3	32 ± 5	4 ± 1		nd	
**Phenolic compounds**												
Phenol [108-95-2]	2	12 ± 2	2 ± 1		nd		11	31 ± 5	11 ± 2		nd	
Cresols [95-48-7, 106-44-5]	2	41 ± 6	10 ± 2		nd		17	92 ± 14	5 ± 1	1	4 ± 1	4 ± 1
Xylenols [95-87-4, 576-26-1]	2	72 ± 11	15 ± 2		nd		17	146 ± 22	12 ± 2		nd	
Ethylmethylphenol [3855-26-3]	2	15 ± 2	2 ± 1	1	1 ± 1	1 ± 1	14	11 ± 2	1 ± 1		nd	

The expanded uncertainty representing the 95% confidence interval is 15%. The expanded uncertainty was calculated using a factor of k = 2. The uncertainty of the results was estimated on the basis of available data, including data on the accuracy of the measurement system used and data on repeatability obtained experimentally. N: number of measurements in which a given compound was identified. max.: the highest identified value. GM: geometric mean value for all measurements. nd: not detected.
